# Lumbar artery pseudoaneurysm following percutaneous nephrolithotripsy: Treatment by transcatheter embolization

**DOI:** 10.4103/0970-1591.42628

**Published:** 2008

**Authors:** Venkat Tummala, Kunal I. Nanavati, Joes M. Yrizarry, Thomas Scagnelli

**Affiliations:** Department of Radiology, section of vascular and interventional radiology, Jackson Memorial Hospital, Miami, Florida, USA

**Keywords:** Embolization, lumbar artery, percutaneous nephrolithotripsy, percutaneous nephrostomy, pseudoaneursym

## Abstract

Vascular complications from percutaneous nephrostomy/nephrolithotripsy (PCN/PCNL) mostly involve the kidneys. Lumbar artery pseudoaneurysms from PCN and PCNL are a rare occurrence. We report a case of lumbar artery pseudoaneurysm following PCNL. This was treated successfully by transcatheter embolization.

## INTRODUCTION

Following percutaneous nephrolithotripsy (PCNL), renal vascular complications ranging from pseudoaneurysms to arteriovenous fistulas have been extensively reported in the literature.[1,2] Lumbar artery pseudoaneurysms following percutaneous nephrostomy (PCN) and PCNL are a rare occurrence. Considerations involved in the diagnosis and management of lumbar artery pseudoaneurysms have been discussed.

## CASE REPORT

**Figure 1:**
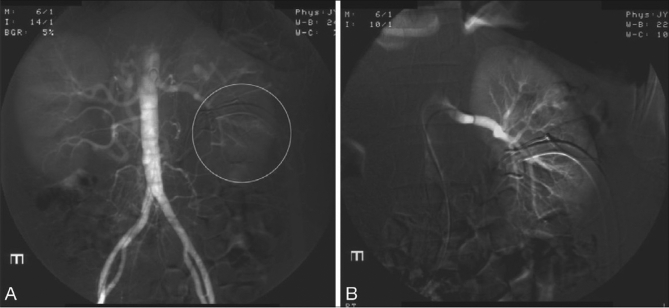
(A) Digital subtraction aortogram with the catheter at the level of renal arteries. Circled area in the flush aortogram shows the region of hypervascularity in relation to the catheter and kidney. (B) Selective left renal angiogram that showed no evidence of renal vascular injury

A 51-year-old male patient presented to the emergency room (ER), two days following an uneventful left PCNL for a recurrent 2.5 cm calculus in the renal pelvis, with a complaint of intermittent bleeding around the Malecot catheter [24 Fr]. Prior to PCNL, the access was provided by interventional radiologists with fluoroscopic guidance through posterior midcalyx. An 8 Fr nephroureteral catheter was placed and the procedure was uneventful. His blood pressure on presentation to ER was 80/60. Hemoglobin/Hematocrit before the procedure, two days prior, was 16/48. Hemoglobin/Hematocrit on presentation to the ER was 13/39. His coagulation profile included PT-12 INR 1.06 PTT-42 that was unchanged from prior to the procedure. (In lieu of his elevated PTT, further laboratory tests before the procedure proved positive for anti-PTT antibody, consistent with lupus anticoagulant antibody.) Patient had a contrast enhanced computed tomography (CT) that showed no active contrast extravasation or perinephric hematoma. Angiography was performed with concern of occult renal vascular injury. Initial abdominal aortogram was performed via a right common femoral arterial approach that showed a suspicious area of hypervascularity in the region of the Malecot catheter none. A selective renal angiogram was then performed that showed no evidence of renal vascular injury. Review of the aortogram at this time raised the suspicion of lumbar arterial branch feeding the hypervascular region [Figure 1A,B].

**Figure 2:**
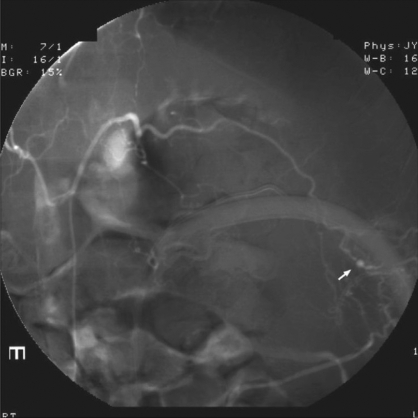
Selective first left lumbar arteriogram showing the pseudoaneursym (arrow) from a branch vessel

**Figure 3:**
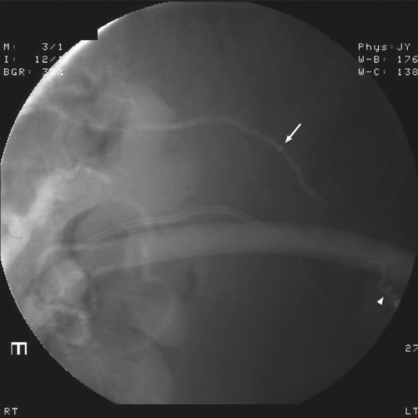
Post-embolization limited arteriogram showing successful exclusion of pseudoaneursym with preservation of flow in the main artery. Arrowhead shows the embolized coils. Arrow shows the tip of the renegade catheter in the main trunk

Selective catheterization of the first left lumbar artery was performed with a 4Fr Cobra catheter (Meditech/Boston-Scientific, Watertown, MA). Lumbar arteriogram thus performed showed a pseudoaneursym from a peripheral branch that was in close proximity to the catheter [Figure 2]. Selective catheterization of this branch was performed with Coaxial Renegade 18 microcatheter (Boston Scientific, Natick, MA), coil embolization with 3 mm × 2 cm coils (Cook, Bloomington, IN) followed by gelfoam pledget embolization with successful exclusion of the feeding branch (one coil was deployed distal and two similar coils were deployed proximal to pseudoaneurysm). Gel foam pledgets were embolized to achieve stasis in the feeding branch as there was evidence of flow despite coil embolization. Final angiogram demonstrated successful exclusion of the pseudoaneurysm with preserved flow in the main trunk of the lumbar artery [Figure 3]. Subsequent to the embolization, patient had no further episodes of bleeding around the catheter. Blood-stained urine, however, persisted which eventually resolved over the next few days, with continuous saline irrigation of the renal collecting system via the Malecot catheter. Patient was discharged home four days after the embolization. He was subsequently seen in Interventional Radiology for a follow-up nephrostogram via the malecot catheter to evaluate for remnant stones [the routine practice at our institution is to remove malecot catheter within two weeks from PCNL]. His catheter was removed without complications following the nephrostogram. He remained asymptomatic with no further episodes of bleeding through the tract following removal of the malecot catheter.

## DISCUSSION

Srivastava *et al*.[3] retrospectively analyzed data of 1854 patients that underwent angiography and/or embolization for bleeding control following percutaneous lithotomy. Twenty-two of 23 vascular lesions involved the renal vasculature. The remaining one lesion involved the lumbar artery, which was incidentally diagnosed on renal angiogram. This series had patients present with severe hematuria. We report this case of unsuspected lumbar artery pseudoaneursym following PCNL that resulted in significant bleeding around the catheter necessitating endovascular management.

Operative control of lumbar artery bleeding is often difficult, since the site of origin may not be readily isolated. Moreover, exploration of the retroperitoneum may compound bleeding by releasing the tamponading effect of the surrounding tissues. Angiographic procedures facilitate not only a fast and minimally invasive diagnosis, but also an immediate therapeutic effect.[4] The lumbar arteries are usually paired arteries arising from the dorsal aspect of the aorta. After arising from the aorta, the lumbar arteries encircle the vertebral bodies and divide into small branches to the psoas muscle and to the radicular medullary artery before dividing into anterior and posterior branches. The anterior branch supplies the quadratus lumborum and sacrospinal muscle, and then branches to the muscles and skin of the flank. The posterior branch supplies the sacrospinalis and skin on the back. Both these branches run in a dorsal relation to the kidney and are thus prone to injury during percutaneous renal procedures.[2] The presence of blood in posterior para-renal space should raise suspicion of lumbar artery injury.

The anatomical and technical considerations are important, especially for procedures involving arterial embolization of the lumbar arteries. In case selective renal angiography fails to find a bleeding source, both aortography and selective angiography of the lumbar arteries at the level of tract or biopsy puncture have to be performed. We routinely perform abdominal aortography before performing selective renal angiography, in an attempt to define renal arterial anatomy before proceeding to selective catheterization. Selective renal angiogram performed in this patient was unremarkable, raising concern of lumbar arterial injury. Selective first left lumbar arteriogram was then obtained that showed the pseudoaneursym from a branch vessel. Particular attention should be given to the origin of great anterior radicular artery [artery of Adamkiewicz] that may arise anywhere from the sixth intercostal to the second lumbar artery. The tip of the catheter should be positioned beyond the spinal branches and catheterization of the feeding branch as close to the pseudoaneursym as possible should be achieved, to avoid spinal cord-related complications from inadvertent non-target embolization. For the same reason, liquid embolic agents for the purpose of embolization in this anatomical location are not recommended. Coil embolization should be performed distally first and then proximal to the pseudoaneurysm so as to prevent the pseudoaneurysm from refilling via collateral supply. Coil embolization may be supplemented with gel foam pledgets as used in this patient to achieve hemostasis after coil embolization for a successful outcome. Too forceful an injection, with the tip of the catheter located close to the origin of the radicular medullary artery should be avoided to prevent spinal cord injury. No neurological complications have occurred following embolization of the lumbar artery pseudoaneursym in this patient.

Patients with bleeding diathesis are more prone to vascular complications from any invasive procedure. This patient had lupus anticoagulant antibody, which although prolongs Partial thromboplastin time (PTT) *in vitro*, in theory, results in thrombosis in small vasculature *in vivo*. It is unlikely that elevated PTT in this particular scenario contributed to his vascular complication.

In conclusion, lumbar artery pseudoaneursym following percutaneous renal procedures is a rare occurrence; however, it may present as a major diagnostic and therapeutic challenge. Presence of blood in the posterior para-renal space on a CT scan should raise suspicion of lumbar artery injury. In cases where aortography and selective renal angiogram fail to identify the source of bleeding, selective lumbar arteriography at the level of tract or puncture should be performed to rule out lumbar arterial injury. Coil embolization supplemented with gel foam pledgets is a safe and effective method of controlling the bleeding, provided the spinal branches are identified and avoided, if any.
